# Artificial Intelligence for Diagnosing Normal Anatomical Variants and Pathological Oral Mucosal Lesions: A Prospective Observational Study

**DOI:** 10.3390/medicina62081610

**Published:** 2026-08-21

**Authors:** Ana Glavina, Marija Galešić, Bojan Poposki, Antonija Tadin

**Affiliations:** 1 Department of Dental Medicine, University Hospital of Split, 21000 Split, Croatia; atadin@mefst.hr; 2 Department of Oral Medicine, Study of Dental Medicine, School of Medicine, University of Split, 21000 Split, Croatia; marija.galesic311@gmail.com; 3 Department of Oral and Periodontal Diseases, Faculty of Dentistry—Skopje, Ss. Cyril and Methodius University in Skopje, 1000 Skopje, North Macedonia; bpoposki@stomfak.ukim.edu.mk; 4 Department of Restorative Dental Medicine and Endodontics, Study of Dental Medicine, School of Medicine, University of Split, 21000 Split, Croatia

**Keywords:** artificial intelligence, mouth mucosa, diagnosis, oral, photography, decision support systems, clinical

## Abstract

*Background and Objectives*: Artificial intelligence (AI) is increasingly used in clinical dentistry, but its diagnostic accuracy for oral mucosal lesions based on clinical photographs remains insufficiently validated. This prospective observational study compared the Top-1 diagnostic accuracy of ChatGPT-4o and ChatGPT-5 in identifying normal anatomical variants and pathological oral mucosal lesions and evaluated their performance across anatomical sites. *Materials and Methods*: Seventy adults with either normal anatomical variants (n = 21) or pathological oral mucosal lesions (n = 49) were consecutively recruited at the Department of Dental Medicine, University Hospital of Split, Croatia. One standardized clinical photograph per patient was analyzed by ChatGPT-4o and ChatGPT-5 under image-only and image-plus-text conditions using identical prompts. The reference diagnosis was established by an oral medicine specialist, with histopathological examination (HPE) performed when clinically indicated. Diagnostic performance was assessed using Top-1 accuracy and McNemar’s test. *Results*: Both models showed low accuracy with image-only input, but performance improved significantly after clinical information was added (*p* < 0.001). Overall Top-1 accuracy increased from 19.0% to 69.0% for ChatGPT-4o and from 9.0% to 51.0% for ChatGPT-5. For normal anatomical variants, accuracy increased from 14.3% to 81.0% and from 14.3% to 76.2%, respectively. For pathological oral mucosal lesions, accuracy increased from 20.4% to 63.3% and from 6.1% to 40.8%, respectively. ChatGPT-4o showed numerically higher accuracy than ChatGPT-5, particularly for pathological oral mucosal lesions, but no statistically significant difference was found between the models in the corresponding paired comparisons. *Conclusions*: Diagnostic performance was limited with image-only input but improved substantially when standardized clinical information accompanied the images. The numerical differences between models, particularly for pathological oral mucosal lesions, may be clinically relevant but do not establish superiority or equivalence. Neither model can currently replace conventional clinical diagnosis, and AI should be regarded as a clinical decision-support tool for evaluating oral mucosal lesions.

## 1. Introduction

Artificial intelligence (AI) has rapidly evolved and is increasingly used in medicine for diagnosis, clinical decision-making, and predicting treatment outcomes [1,2]. In dentistry, advances in machine learning and medical imaging have enabled the analysis of large volumes of clinical, radiographic, and patient data, supporting more accurate diagnosis and treatment planning [1]. AI offers several potential advantages, including improved diagnostic precision, personalized treatment planning, and greater efficiency through automation. However, important challenges remain, including bias, limited reliability, privacy concerns, and the lack of human judgment and empathy, which continue to limit its role as an independent tool for clinical decision-making [3].

AI has been increasingly applied across various fields of dentistry, including the diagnosis of dental caries, periodontology, orthodontics, and implant dentistry. Machine learning–based systems have shown promising results in detecting carious lesions from clinical photographs and imaging modalities, supporting diagnostic decision-making [4,5]. In periodontology, AI algorithms have been used for radiographic interpretation, assessment of alveolar bone loss, and treatment planning, improving periodontal diagnosis and patient management [1,4,6]. In orthodontics, deep learning models have demonstrated high accuracy in cephalometric analysis and have been applied to support treatment planning and predict tooth movement [4,7]. Similarly, in implant dentistry, AI supports treatment planning through the identification of anatomical structures on cone-beam computed tomography (CBCT) images and implant identification, potentially improving diagnostic accuracy and clinical decision-making [8,9,10,11]. Beyond these applications, AI has also shown promising potential in oral oncology. According to the latest Global Cancer Observatory (GLOBOCAN) estimates from the International Agency for Research on Cancer (IARC), cancers of the lip and oral cavity account for approximately 390,000 new cases annually worldwide, underscoring the importance of early detection for improving treatment outcomes and survival [12,13]. AI-based systems have been developed to detect head and neck tumors using medical imaging and have demonstrated the ability to recognize malignant transformation in potentially malignant oral lesions, including leukoplakia and lichenoid lesions [1,4,14]. Deep learning approaches have also shown strong performance in detecting metastatic lymph node involvement [15].

Recent advances in large language models (LLMs) have expanded the potential applications of AI in clinical medicine. Multimodal LLMs, capable of processing both visual and textual information, may provide additional value in medical image interpretation by integrating clinical context with visual findings [16]. Preliminary studies have shown promising results in the assessment of oral and maxillofacial conditions. However, evidence regarding their diagnostic accuracy for oral mucosal lesions remains limited [17]. In addition, the impact of adding standardized clinical information to image-based assessments and the comparative performance of different LLM versions require further investigation.

The primary objective of this study was to evaluate and compare the Top-1 diagnostic accuracy of two AI platforms, ChatGPT-4o and ChatGPT-5, in identifying normal anatomical variants and pathological oral mucosal lesions under image-only and image-plus-text conditions. Secondary objectives were to assess diagnostic performance across different anatomical sites and to describe the clinical and demographic distribution of the included oral mucosal conditions. We hypothesized that adding standardized clinical information would improve the diagnostic accuracy of both AI models and that diagnostic performance would differ between the two platforms. The reference-standard diagnosis was established through conventional clinical examination and supplemented with histopathological confirmation when indicated.

## 2. Materials and Methods

### 2.1. Study Design, Subjects, Inclusion and Exclusion Criteria

This prospective observational study was conducted from 25 February to 25 August 2025, at the Department of Dental Medicine, University Hospital of Split, Split, Croatia, in accordance with the principles of the Declaration of Helsinki [18]. The study was designed in line with the Standards for Reporting Diagnostic Accuracy Studies—Artificial Intelligence (STARD-AI) recommendations for diagnostic accuracy studies incorporating AI [19]. Consecutive eligible patients attending the Department of Dental Medicine during the study period were invited to participate. A total of 70 patients (33 male and 37 female), aged 18–87 years, were enrolled. For each case, the reference clinical diagnosis was established by an oral medicine specialist with more than five years of clinical experience (A.G.), based on conventional clinical examination and diagnostic criteria appropriate for each condition. Histopathological confirmation was obtained for lesions that underwent biopsy. Of the 49 pathological oral mucosal lesions included in the study, 30 (61.2%) underwent biopsy and were histopathologically confirmed, including 17 cases of oral lichen planus (OLP), 1 case of pigmentation, 1 case of linear IgA disease (LAD), 2 cases of oral squamous cell carcinoma (OSCC), 8 cases of pemphigus vulgaris (PV), and 1 case of bullous pemphigoid (BP). The remaining 19 pathological oral mucosal lesions (38.8%) were diagnosed clinically based on conventional oral examination and established diagnostic criteria appropriate for each condition. Clinical diagnoses were based on characteristic clinical appearance, anatomical distribution, patient history, and relevant clinical findings. These conditions included friction-induced hyperkeratosis, minor aphthae, median rhomboid glossitis, acute pseudomembranous candidiasis, chronic hyperplastic candidiasis, prosthetic stomatitis, nicotine melanosis, drug-induced gingival hyperplasia, mucocele, hemangioma, nicotine stomatitis, amalgam-associated lichenoid reaction, and oral manifestations associated with Crohn’s disease. Histopathological examination was not performed when the clinical presentation and established diagnostic criteria were considered sufficient for diagnosis and biopsy was not clinically indicated for diagnosis or management. Histopathological confirmation was obtained for lesions for which biopsy was clinically indicated. A complete list of the reference diagnoses, their frequencies, and histopathological confirmation status is provided in Supplementary Tables S1 and S2. For each participant, demographic data (age and sex) and the anatomical location of the lesion (lips, buccal mucosa, vestibule, gingiva, tongue, floor of the mouth, or palate) were recorded. Sex was recorded as a demographic variable, and sex-stratified analyses were considered exploratory. Exclusion criteria were applied to minimize factors that could influence the clinical appearance of the oral mucosa and potentially affect AI-based diagnostic performance. The study protocol was approved by the Ethics Committee of the University Hospital of Split, Split, Croatia, on 20 February 2025 (Class 520-03/25-01/60; Reg. No. 2181-147/01-06/LJ.Z.-25-02).

The inclusion criterion was as follows:

1. Adults (≥18 years) presenting with clinically diagnosed normal anatomical variants or pathological oral mucosal lesions.

The exclusion criteria were as follows:

1. Incomplete clinical records;
2. Clinical photographs with inadequate image quality (e.g., poor focus, motion blur, insufficient lighting, or incomplete visualization of the lesion) that rendered them unsuitable for AI analysis;
3. Prior treatment of the lesion before image acquisition;
4. Use of systemic corticosteroids, immunosuppressive agents, chemotherapy, or other medications known to induce oral mucosal alterations within the previous 12 months;
5. Previous oral surgical procedures involving the affected anatomical site;
6. Refusal or inability to provide informed consent.

The study population consisted of consecutive adult patients treated at a single tertiary referral center. Consequently, the sample may not fully represent the broader population of patients with oral mucosal lesions encountered in primary care or other healthcare settings. Therefore, the findings should be interpreted within the context of this study population, and their generalizability to other populations and clinical environments should be confirmed in larger, multicenter studies.

### 2.2. Image Collection and Processing

This prospective observational study included 70 adult patients who presented with either normal anatomical variants (n = 21) or pathological oral mucosal lesions (n = 49) at different anatomical sites during their first or follow-up examination at the Department of Dental Medicine, University Hospital of Split, Split, Croatia. One to three clinical photographs were obtained from each patient using the same smartphone camera under routine clinical conditions. When more than one photograph was available, two investigators (A.G. and A.T.) independently reviewed the images and, by consensus, selected a single representative photograph based on image quality (focus, sharpness, and illumination), complete visualization of the lesion, and the absence of artifacts or obstructions. The images were then processed using Adobe Photoshop (Adobe Inc., San Jose, CA, USA). All photographs were resized to a standardized resolution of 1024 × 768 pixels, cropped to center the lesion, and had irrelevant background elements removed to minimize non-diagnostic visual information while preserving clinically relevant lesion characteristics. No color correction, enhancement, or other modifications that could alter lesion morphology or diagnostic features were applied. To ensure patient anonymity, potentially identifiable facial features were obscured before analysis. All clinical photographs were anonymized before upload to the AI platform, and no patient identifiers or other personally identifiable information were provided during AI analysis. The images were analyzed using the publicly available ChatGPT web interface solely to evaluate diagnostic performance. A total of 70 representative clinical photographs (one image per patient) were included in the AI analysis. Both ChatGPT-4o and ChatGPT-5 were evaluated using the same set of standardized, representative clinical images and an identical prompt. Each image was analyzed in a separate, newly initiated chat session to prevent contextual carryover and ensure independent assessments. The presentation order and testing conditions were identical for both models.

### 2.3. AI Platforms

Two large language and multimodal AI models, ChatGPT-4o and ChatGPT-5 (OpenAI, San Francisco, CA, USA), were evaluated in this study. ChatGPT-4o has been described as a multimodal model capable of processing text and images [20], whereas ChatGPT-5 represents a next-generation model developed by OpenAI [21]. Both models were accessed through the official ChatGPT web interface and evaluated under identical experimental conditions during the study period. No fine-tuning, customization, external plugins, or additional tools were applied. The default settings available through the ChatGPT interface were used, and no user-adjustable generation parameters (e.g., temperature or sampling settings) were modified. Because the models were evaluated through the web interface rather than an application programming interface (API), specific API snapshots and underlying generation parameters were not available. Memory functionality was disabled, and each image was analyzed in a newly initiated chat session to prevent contextual carryover between cases. Responses were generated once per case without regeneration or manual selection. Both models received identical prompts and standardized clinical information, as described in Section 2.4.

### 2.4. Diagnostic Process Using AI

This study used a within-subject experimental design in which all 70 clinical images were evaluated by two AI models (ChatGPT-4o and ChatGPT-5) under two experimental conditions: image-only analysis and image-plus-text analysis. In the image-only condition, each model received a standardized clinical photograph and the following prompt: “Based on the provided clinical image, please provide the most likely diagnosis of the oral mucosal condition. List your answer and include possible differential diagnoses, if applicable.” In the image-plus-text condition, each image was accompanied by a standardized, one-sentence clinical description, and the models were given the following prompt: “Based on the provided clinical image and accompanying clinical description, please provide the most likely diagnosis of the oral mucosal condition. List your answer and include possible differential diagnoses, if applicable.” The accompanying clinical descriptions were prepared using a predefined template to ensure consistency across cases. The template structure was as follows: “A [age]-year-old [sex] patient presents with a [single/multiple] [color] lesion(s) located on the [anatomical site], present for [duration], associated with [symptoms, if present]. Clinical characteristics include [surface characteristics, presence/absence of ulceration, and approximate size].” The descriptions were derived from available clinical records and standardized before AI evaluation. No additional diagnostic interpretation or disease-specific terminology was added. An example of the standardized clinical description is: “A 45-year-old woman presents with multiple erythematous lesions with irregular whitish borders located on the dorsum of the tongue, present for two weeks and associated with a burning sensation. The lesions are characterized by a nonulcerated surface and involve multiple areas.”

All evaluations were performed independently, with each case analyzed in a newly initiated chat session to prevent contextual carryover. Only anonymized clinical photographs were uploaded, without patient identifiers or any other personally identifiable information. Diagnostic performance was assessed using strict Top-1 accuracy, defined as the proportion of cases in which the first (primary) diagnosis generated by the AI matched the reference-standard diagnosis. Although the models were instructed to provide differential diagnoses when appropriate, only the primary diagnosis was included in the accuracy assessment, and diagnoses listed solely as differential possibilities were not counted as correct.

AI-generated responses were evaluated by a single investigator (A.G.), who was blinded to both the identity of the AI model (ChatGPT-4o or ChatGPT-5) and the experimental condition (image-only or image-plus-text). The first (primary) diagnosis generated by each model was compared with the reference-standard diagnosis using predefined matching criteria. Minor differences in terminology and widely accepted synonymous diagnostic terms referring to the same clinical entity were considered equivalent, whereas broader diagnostic categories, related conditions, and diagnoses listed only as differential possibilities were not considered correct. The matching criteria were defined before data analysis and applied consistently across all cases. All AI analyses were performed on 30 August 2025, after completion of patient recruitment and image collection. Both ChatGPT-4o and ChatGPT-5 were evaluated using the same finalized dataset, standardized prompts, and identical testing conditions. The standard, default user-accessible configuration available in the ChatGPT interface was used, and no additional reasoning (“Thinking”) mode or custom settings were enabled.

### 2.5. Statistical Analysis

Data were entered and coded in Microsoft Excel (version 16.0; Microsoft Corporation, Redmond, WA, USA) and analyzed using IBM SPSS Statistics (version 23.0; IBM Corp., Armonk, NY, USA). Diagnostic performance was assessed using Top-1 accuracy, defined as the proportion of cases in which the first diagnosis provided by the AI model matched the reference standard diagnosis. Diagnostic accuracy between AI models (ChatGPT-4o vs. ChatGPT-5) and experimental conditions (image-only vs. image-plus-text) was compared using McNemar’s test for paired proportions. For all accuracy estimates, 95% confidence intervals (CIs) were calculated using bootstrap resampling with 1000 iterations. Anatomical site-specific analyses were reported descriptively because several subgroups included only a limited number of observations; therefore, these results should be interpreted as exploratory. A *p*-value < 0.05 was considered statistically significant. The sample size was based on a paired comparison design using McNemar’s test. A total of 70 cases provided >80% statistical power to detect the prespecified effect under the assumed paired-proportion parameters, with an alpha level of 0.05. Because reliable prior estimates of baseline diagnostic accuracy and paired discordance rates for AI-based diagnosis of oral mucosal lesions were not available, these parameters were based on assumptions used for the power calculation and were not derived from established external estimates.

## 3. Results

### 3.1. Participant Characteristics

This prospective observational study included 70 participants aged 18–87 years (mean age, 47.7 ± 20.7 years). Of these, 21 (30.0%) had normal anatomical variants and 49 (70.0%) had pathological oral mucosal lesions. Overall, 33 participants (47.1%) were male and 37 (52.9%) were female.

The demographic and clinical characteristics of participants with normal anatomical variants are summarized in Table 1. The mean age in this group was 36.3 ± 20.0 years, and females accounted for 61.9% (n = 13) of cases. The tongue was the most common site of normal anatomical variants (n = 11, 52.4%), followed by the buccal mucosa (n = 6, 28.6%). One lesion each (4.8%) was identified on the lip, vestibular mucosa, palate, and gingiva, and no normal anatomical variants were observed on the floor of the mouth. The tongue was also the most frequently affected site in both males (n = 4, 50.0%) and females (n = 7, 53.8%). Because of the limited number of cases in some anatomical subgroups, site-specific analyses should be interpreted as exploratory and descriptive.

Table 2 summarizes the demographic characteristics and anatomical distribution of participants with pathological oral mucosal lesions. This group included 49 participants (25 males [51.0%] and 24 females [49.0%]) with a mean age of 52.6 ± 19.2 years. The gingiva was the most frequently affected site (n = 11, 22.4%), followed by the lips (n = 10, 20.4%), the tongue and buccal mucosa (n = 8 each, 16.3%), the palate (n = 7, 14.3%), the vestibular mucosa (n = 3, 6.1%), and the floor of the mouth (n = 2, 4.1%). Among male participants, lesions were most commonly located on the lips (n = 6, 24.0%), whereas gingival lesions were most frequent among female participants (n = 6, 25.0%). Because of the limited number of cases in some anatomical subgroups, these distributions should be interpreted descriptively.

### 3.2. Top-1 Diagnostic Accuracy by Anatomical Site

Table 3 summarizes the Top-1 diagnostic accuracy of ChatGPT-4o and ChatGPT-5 for normal anatomical variants by anatomical site. In the image-only condition, both models correctly identified 3 of 21 normal anatomical variants (14.3%) as the primary diagnosis. Adding standardized clinical descriptions substantially improved Top-1 diagnostic accuracy, with ChatGPT-4o correctly identifying 17 cases (81.0%) and ChatGPT-5 correctly identifying 16 cases (76.2%). Among the evaluated anatomical sites, tongue lesions showed the highest observed Top-1 diagnostic accuracy. In the image-only condition, both models correctly identified 3 of 11 tongue lesions (27.3%), whereas in the image-plus-text condition, both models correctly identified 10 of 11 tongue lesions (90.9%) as the primary diagnosis. These site-specific findings should be interpreted cautiously because of the limited number of cases in some anatomical subgroups.

Table 4 summarizes the Top-1 diagnostic accuracy of ChatGPT-4o and ChatGPT-5 for pathological oral mucosal lesions by anatomical site. In the image-only condition, ChatGPT-5 correctly identified 3 of 49 pathological oral mucosal lesions (6.1%) as the primary diagnosis, whereas ChatGPT-4o correctly identified 10 of 49 lesions (20.4%). After standardized clinical information was added, Top-1 diagnostic accuracy improved, with ChatGPT-5 correctly identifying 20 of 49 cases (40.8%) and ChatGPT-4o correctly identifying 31 of 49 cases (63.3%) as the primary diagnosis. Among the evaluated anatomical sites, lip lesions showed the highest Top-1 diagnostic accuracy. In the image-only condition, ChatGPT-5 correctly identified 3 of 10 lip lesions (30.0%), increasing to 5 of 10 cases (50.0%) in the image-plus-text condition. For ChatGPT-4o, accuracy for lip lesions increased from 4 of 10 cases (40.0%) in the image-only condition to 8 of 10 cases (80.0%) after standardized clinical information was added. These site-specific findings should be interpreted cautiously because of the limited number of cases in some anatomical subgroups.

### 3.3. Overall Top-1 Diagnostic Accuracy for Normal Anatomical Variants and Pathological Oral Mucosal Lesions

**Figure 1:**
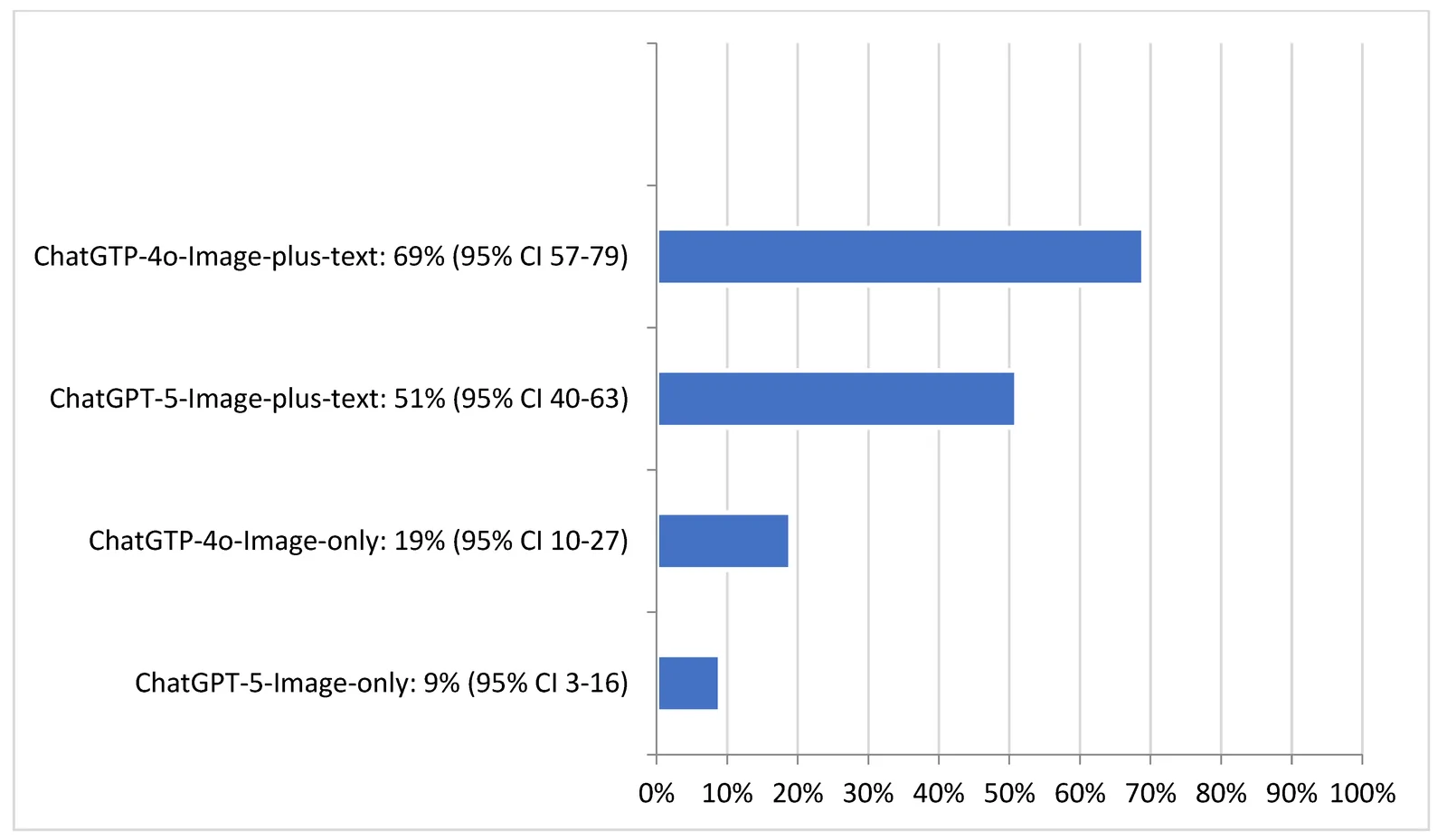
Overall Top-1 diagnostic accuracy of ChatGPT-4o and ChatGPT-5 for oral mucosal changes (n = 70) under image-only and image-plus-text conditions. The *x*-axis represents the evaluated AI model and input condition (ChatGPT-5 image-only, ChatGPT-4o image-only, ChatGPT-5 image-plus-text, and ChatGPT-4o image-plus-text). The *y*-axis represents Top-1 diagnostic accuracy (%). Bars represent the proportion of cases in which the first diagnosis generated by the AI model matched the reference-standard diagnosis. Error bars indicate 95% confidence intervals (CIs).

Figure 1 summarizes the overall Top-1 diagnostic accuracy of ChatGPT-4o and ChatGPT-5 for all oral mucosal changes (normal variants and pathological, N = 70). A statistically significant improvement in Top-1 diagnostic accuracy was observed for both models when standardized clinical information was added to the clinical photographs (*p* < 0.001). For ChatGPT-5, Top-1 accuracy increased from 9.0% in the image-only condition to 51.0% in the image-plus-text condition. For ChatGPT-4o, Top-1 accuracy increased from 19.0% in the image-only condition to 69.0% in the image-plus-text condition. Although ChatGPT-4o showed numerically higher Top-1 accuracy than ChatGPT-5 in both experimental conditions, particularly in the image-plus-text condition (69.0% vs. 51.0%), the differences between the models were not statistically significant in the paired comparisons.

#### 3.3.1. Top-1 Diagnostic Accuracy for Normal Anatomical Variants

**Figure 2:**
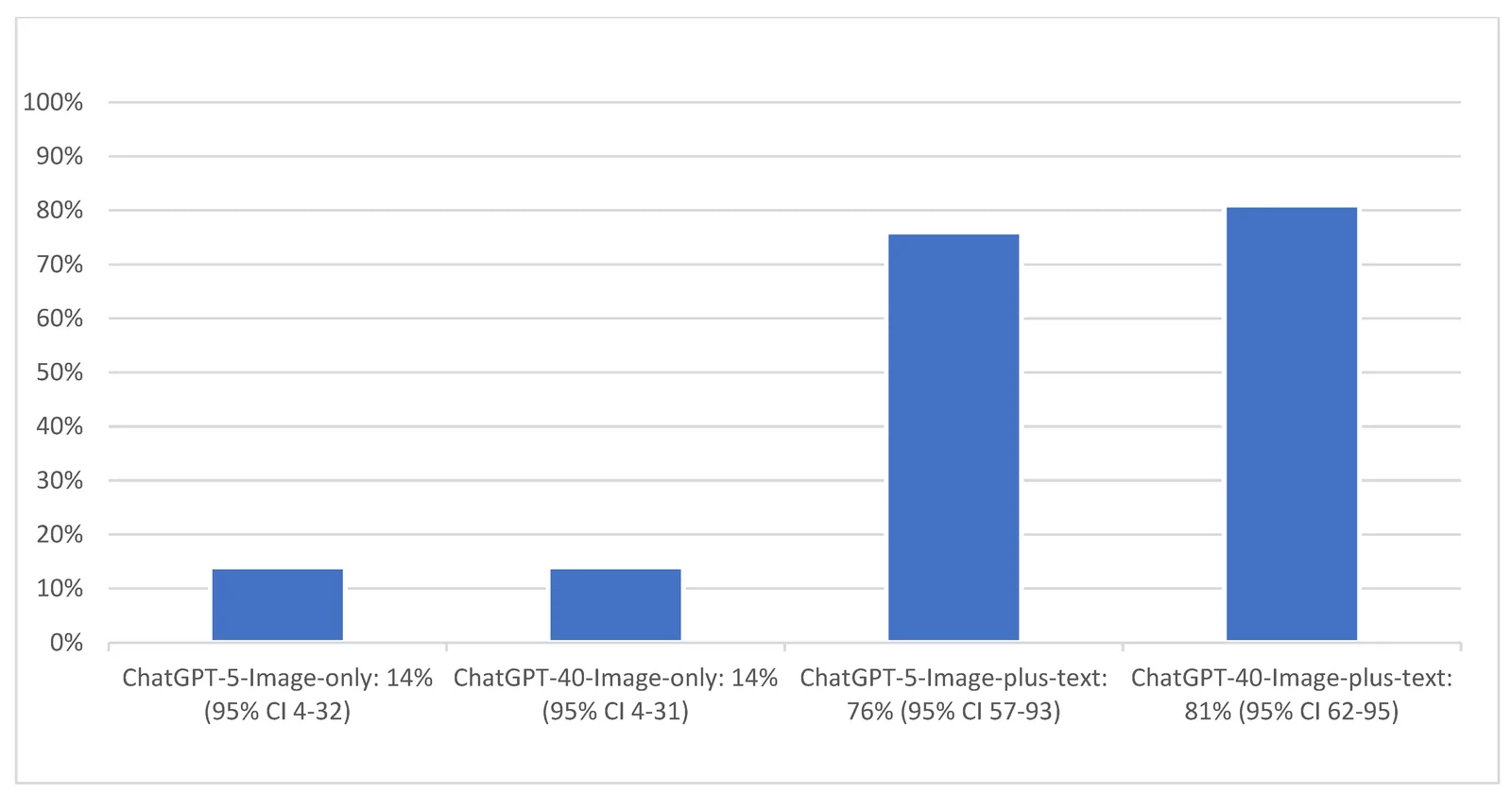
Top-1 diagnostic accuracy of ChatGPT-4o and ChatGPT-5 for normal anatomical variants (n = 21) under image-only and image-plus-text conditions. The *x*-axis represents the evaluated AI model and input condition (ChatGPT-5 image-only, ChatGPT-4o image-only, ChatGPT-5 image-plus-text, and ChatGPT-4o image-plus-text). The *y*-axis represents Top-1 diagnostic accuracy (%). Bars represent the proportion of cases in which the first diagnosis generated by the AI model matched the reference-standard diagnosis. Error bars indicate 95% confidence intervals (CIs).

A statistically significant improvement in Top-1 diagnostic accuracy was observed between the image-only and image-plus-text conditions for both AI models (*p* < 0.001). For ChatGPT-5, Top-1 accuracy for normal anatomical variants increased from 14.3% in the image-only condition to 76.2% in the image-plus-text condition. Similarly, ChatGPT-4o achieved a Top-1 accuracy of 14.3% in the image-only condition, which increased to 81.0% with the addition of standardized clinical information (Figure 2).

#### 3.3.2. Top-1 Diagnostic Accuracy for Pathological Oral Mucosal Lesions

**Figure 3:**
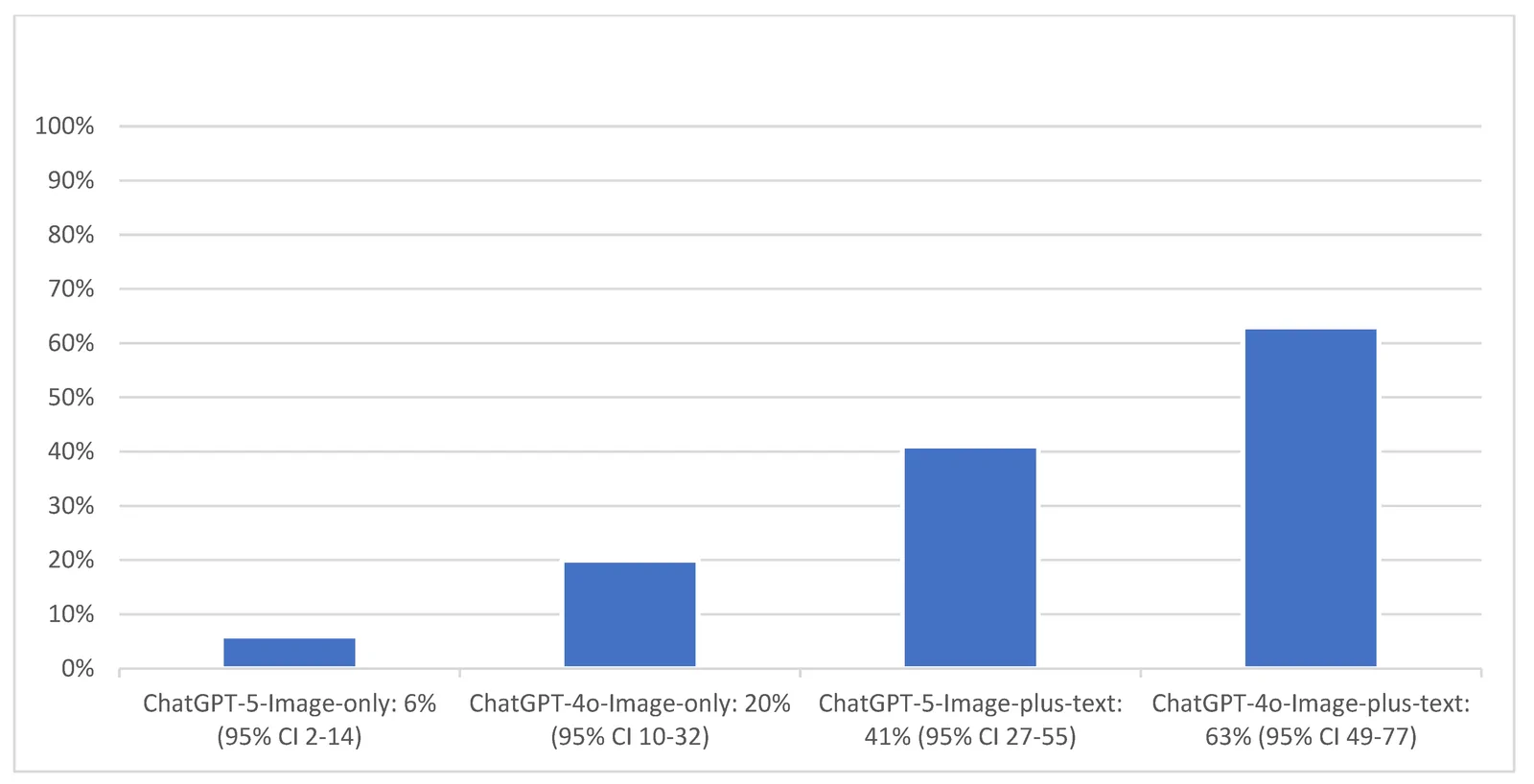
Top-1 diagnostic accuracy of ChatGPT-4o and ChatGPT-5 for pathological oral mucosal lesions (n = 49) under image-only and image-plus-text conditions. The *x*-axis represents the evaluated AI model and input condition (ChatGPT-5 image-only, ChatGPT-4o image-only, ChatGPT-5 image-plus-text, and ChatGPT-4o image-plus-text). The *y*-axis represents Top-1 diagnostic accuracy (%). Bars represent the proportion of cases in which the first diagnosis generated by the AI model matched the reference-standard diagnosis. Error bars indicate 95% confidence intervals (CIs).

A statistically significant improvement in Top-1 diagnostic accuracy was observed for both AI models after standardized clinical information was added (*p* < 0.001). ChatGPT-5 achieved a Top-1 accuracy of 6.1% for pathological oral mucosal lesions in the image-only condition, which increased to 40.8% in the image-plus-text condition. Similarly, ChatGPT-4o achieved a Top-1 accuracy of 20.4% in the image-only condition, which increased to 63.3% in the image-plus-text condition after standardized clinical information was added (Figure 3).

### 3.4. Comparison of Top-1 Diagnostic Accuracy for Normal Anatomical Variants and Pathological Oral Mucosal Lesions

No statistically significant differences in Top-1 diagnostic accuracy between normal anatomical variants and pathological oral mucosal lesions were observed for ChatGPT-5 in the image-only condition (*p* = 0.270), ChatGPT-4o in the image-only condition (*p* = 0.553), or ChatGPT-4o in the image-plus-text condition (*p* = 0.148). In contrast, in the image-plus-text condition, ChatGPT-5 demonstrated significantly higher Top-1 diagnostic accuracy for normal anatomical variants [76.2% (95% CI, 57.0–93.0%)] than for pathological oral mucosal lesions [40.8% (95% CI, 27.0–55.0%)] (*p* = 0.006) (Figure 2 and Figure 3). Review of misclassified cases indicated that diagnostic errors occurred across multiple categories of oral mucosal conditions. These errors were heterogeneous, involving both normal anatomical variants and pathological oral mucosal lesions, and no single lesion category appeared to account for a predominant proportion of misclassifications. However, the clinical significance of individual errors varied according to the underlying condition, particularly when potentially malignant or malignant lesions were misclassified.

## 4. Discussion

The primary objective of this prospective comparative study was to evaluate and compare the diagnostic accuracy of two AI platforms, ChatGPT-4o and ChatGPT-5, in recognizing normal anatomical variants and pathological oral mucosal lesions. The findings contribute to the growing body of evidence on the potential role of LLMs in supporting oral medicine diagnostics. Although AI has shown promising applications in medical diagnostics, including the analysis of computed tomography (CT) scans, radiographic images, and clinical photographs of dermatologic and oral diseases [22,23], current AI systems have not yet achieved diagnostic reliability comparable to that of experienced clinicians and therefore should not be considered independent diagnostic tools [24]. These observations align with the findings of Vaira L.A. et al., who reported that ChatGPT-4o demonstrated promising diagnostic performance and clinical reasoning in the assessment of oral mucosal lesions but emphasized that, due to false-negative results, it should currently be regarded as a clinical decision-support tool rather than a standalone diagnostic system [17]. In the present study, ChatGPT-4o showed numerically higher diagnostic accuracy than ChatGPT-5 across the overall sample and in most subgroup analyses. The numerical differences were particularly pronounced in the image-plus-text condition, with an overall Top-1 accuracy of 69.0% for ChatGPT-4o versus 51.0% for ChatGPT-5 and accuracy for pathological oral mucosal lesions of 63.3% versus 40.8%, respectively. However, no statistically significant differences were detected between the two models in the corresponding paired comparisons. These findings should therefore not be interpreted as evidence of equivalence between the models or as evidence of superiority of either model. Rather, the observed numerical differences, particularly for pathological oral mucosal lesions, may be clinically relevant and warrant further investigation in larger, adequately powered comparative studies. Independent validation remains necessary before either model can be considered for routine clinical implementation.

Both AI platforms evaluated in this study are LLMs that have recently evolved into multimodal systems capable of processing both textual and visual information [25,26]. Although previous reports have shown encouraging LLM performance in supporting clinical decision-making [27,28], evidence regarding their diagnostic accuracy in interpreting clinical photographs of oral mucosal lesions remains limited and requires further validation.

One of the principal findings of this study was that adding standardized clinical information to clinical photographs significantly improved the Top-1 diagnostic accuracy of both AI models compared with image-only analysis (*p* < 0.001). These results highlight the importance of combining visual information with relevant clinical context when using LLMs for diagnostic support. In the image-plus-text condition, ChatGPT-5 achieved significantly higher Top-1 diagnostic accuracy for normal anatomical variants than for pathological oral mucosal lesions (76.2% vs. 41.8%; *p* = 0.006), whereas no such difference was observed for ChatGPT-4o. Although ChatGPT-4o demonstrated numerically higher diagnostic accuracy than ChatGPT-5, particularly for pathological oral mucosal lesions, direct comparisons between the two models did not reveal a statistically significant advantage for either platform. Overall, these findings emphasize the importance of incorporating relevant clinical information when using LLMs for diagnostic support, while potential differences between model versions should not be disregarded solely on the basis of non-significant statistical comparisons.

Normal anatomical variants and pathological oral mucosal lesions showed distinct anatomical distributions, with normal anatomical variants occurring most frequently on the tongue and pathological oral mucosal lesions predominantly affecting the gingiva and lips. Diagnostic performance varied across anatomical sites. However, site-specific estimates should be interpreted cautiously because several locations were represented by only a small number of cases. Differences in performance may reflect variations in lesion morphology and image-related factors, such as lighting conditions, shadows, and the presence of blood. Similar findings were reported by Yu S et al., who showed that AI performance varied according to lesion location, with higher accuracy for anatomical sites more frequently represented in the training dataset [29]. These findings highlight the need for larger and more diverse annotated image datasets to improve the generalizability of AI models, particularly for uncommon or atypical clinical presentations.

From a clinical perspective, our findings indicate that image-only input provides limited diagnostic accuracy for assessing oral mucosal lesions, whereas adding standardized clinical information significantly improves diagnostic performance. However, this improvement should be interpreted as the combined effect of image and clinical text inputs, as the independent contribution of textual information could not be determined due to the absence of a text-only control condition. Therefore, the observed increase in accuracy cannot be attributed exclusively to enhanced visual diagnostic capability. Nevertheless, AI should currently be regarded as a clinical decision-support tool rather than a substitute for professional judgment. Its potential role lies in assisting clinicians by supporting differential diagnosis and clinical decision-making, while the final diagnosis should continue to rely on comprehensive clinical examination and established diagnostic procedures. These findings are consistent with those of Chiesa-Estomba C.M. et al., who reported limited performance of ChatGPT-4o in video analysis, suggesting that diagnostic reasoning may depend substantially on accompanying clinical information rather than on visual data alone [28].

Although ChatGPT-4o achieved numerically higher diagnostic accuracy than ChatGPT-5 in most analyses, particularly in the image-plus-text condition, no statistically significant difference was detected between the two models in the corresponding paired comparisons. ChatGPT-5 demonstrated significantly higher diagnostic accuracy for normal anatomical variants than for pathological oral mucosal lesions, whereas no such difference was observed for ChatGPT-4o. These findings suggest that the relationship between lesion type and diagnostic performance may vary across AI models. However, the observed numerical differences between the models, particularly for pathological oral mucosal lesions, may be clinically relevant and warrant further investigation in larger, adequately powered comparative studies. In this study, diagnostic accuracy was assessed using a Top-1 approach, in which only the first diagnosis generated by the AI model was counted as correct. This strict criterion reflects the model’s primary diagnostic output rather than its ability to include the correct diagnosis within a broader differential diagnosis. Although differential diagnoses may provide insight into diagnostic reasoning, their inclusion should be interpreted as diagnostic consideration rather than confirmed diagnostic accuracy. Future studies should incorporate additional standardized, rank-based metrics to further characterize AI diagnostic performance.

Future development and validation of AI models for oral medicine should include evaluation using diverse image-plus-text datasets that reflect the variability of real-world clinical practice. The diagnostic performance of multimodal AI systems may depend not only on image quality but also on the type, completeness, and clinical relevance of the accompanying textual information. Therefore, standardized yet diverse clinical descriptions, including patient characteristics, lesion morphology, anatomical location, symptoms, and clinical history, should be incorporated into future validation studies to better assess model robustness and generalizability. Successful implementation of AI-assisted diagnostics in oral medicine will also require close interdisciplinary collaboration among clinicians, computer scientists, and AI developers. Clinicians provide essential expertise on disease presentation and diagnostic priorities, while computer scientists and AI developers contribute expertise in model optimization, validation strategies, and system integration. Such collaborative efforts are crucial for developing reliable, clinically relevant, and ethically responsible AI tools that support decision-making in oral health care.

The present study has several limitations. First, the relatively small sample size and uneven distribution of lesions across anatomical sites reduced the statistical power of subgroup analyses. Several anatomical locations were represented by only a few cases, resulting in wide CIs and unstable estimates. Therefore, site-specific accuracy values, including extreme values such as 0% or 100%, should be interpreted with caution. These analyses should be considered exploratory and hypothesis-generating rather than definitive estimates of model performance. Future studies with larger, more balanced, externally validated datasets are needed to further evaluate AI performance across different lesion types and anatomical locations. Second, this study was conducted at a single academic dental center and included a heterogeneous spectrum of normal anatomical variants and pathological oral mucosal lesions. Although this reflects clinical variability, the sample may not fully represent broader patient populations, and multicenter studies are needed to confirm the generalizability of these findings. Third, the reference diagnosis was established by a single oral medicine specialist based on conventional clinical examination, with 30 of 49 pathological oral mucosal lesions undergoing biopsy and histopathological confirmation. The remaining 19 pathological oral mucosal lesions were diagnosed clinically using established diagnostic criteria for each condition, and biopsy was not performed when it was not considered clinically indicated, based on the clinical presentation and diagnostic requirements for the individual condition. Consequently, not all pathological oral mucosal lesions were confirmed histopathologically, and inter-rater agreement could not be assessed. Although this approach reflects routine clinical practice in oral medicine, it may have introduced reference standard bias. In addition, AI-generated responses were evaluated by a single blinded investigator, and inter-rater agreement for response adjudication was not assessed. An independent blinded assessment by a second specialist was not performed and represents an additional limitation of the study. Future studies should incorporate blinded assessment by multiple independent experts and, when clinically appropriate, histopathological confirmation to strengthen the reference standard and improve the robustness of response adjudication.

Fourth, diagnostic performance was assessed using Top-1 accuracy, in which only the primary diagnosis generated by the AI model was considered correct. Although this provides a strict measure of agreement with the reference diagnosis, it does not fully capture the potential value of AI-assisted differential diagnosis, because inclusion of a diagnosis in a differential list reflects diagnostic consideration rather than confirmed accuracy. Future studies should evaluate additional standardized, ranking-based metrics. Furthermore, the absence of a text-only control condition prevented isolation of the independent contributions of clinical information and image analysis. Therefore, the observed improvement with image-plus-text input should be interpreted as the combined effect of multimodal information rather than as evidence of enhanced visual diagnostic capability alone. Future studies incorporating image-only, text-only, and image-plus-text conditions are needed to clarify the contribution of each modality. The study did not include a human comparator group and, therefore, cannot determine whether the evaluated AI models provide performance comparable to clinicians with different levels of experience, such as general dentists, residents, or oral medicine specialists. Future studies should incorporate blinded comparisons across different clinician groups to better evaluate the potential role of AI as a diagnostic decision-support tool. Because of the heterogeneous, multiclass nature of oral mucosal conditions and the limited number of cases within individual diagnostic categories, additional performance measures such as sensitivity, specificity, predictive values, and confusion matrices were not considered reliable for the present dataset. Larger, more balanced datasets should include these measures to provide a more comprehensive assessment of AI diagnostic performance.

Repeatability testing with repeated model queries was not performed. Because LLMs may show variability in generated responses and may undergo version updates over time, future studies should include repeated evaluations, clearly documented model versions, and standardized testing protocols to assess the temporal stability and reproducibility of performance. Furthermore, although the evaluated cases were selected from real clinical practice, the possibility that certain typical or textbook-like clinical presentations overlap with patterns represented in the model training data cannot be excluded. External validation using independent datasets is therefore required to confirm the generalizability of these findings. Finally, the AI analyses were conducted using a publicly available web-based AI platform. Although all clinical images were anonymized before upload and no identifiable patient information was provided, the use of a public AI service represents a potential limitation for data governance and privacy. Future studies should consider using institutionally governed AI platforms specifically designed for clinical and research applications.

## 5. Conclusions

This study showed that providing standardized clinical information alongside clinical photographs was associated with improved Top-1 diagnostic accuracy of multimodal LLMs for assessing oral mucosal lesions under the evaluated conditions. Both ChatGPT-4o and ChatGPT-5 demonstrated a statistically significant increase in diagnostic performance when image-plus-text input was provided compared with image-only input. Although ChatGPT-4o achieved numerically higher diagnostic accuracy than ChatGPT-5 across the overall sample and in several subgroup analyses, particularly for pathological oral mucosal lesions, no statistically significant difference was detected between the two models in the corresponding paired comparisons. The observed numerical differences, particularly for pathological oral mucosal lesions, may nonetheless be clinically relevant and warrant further investigation in larger, adequately powered comparative studies.

These findings suggest that relevant clinical information may play an important role in AI-assisted assessment of oral mucosal conditions. ChatGPT-5 demonstrated significantly higher Top-1 diagnostic accuracy for normal anatomical variants than for pathological oral mucosal lesions, whereas no statistically significant difference was observed for ChatGPT-4o between these lesion categories. Diagnostic performance also varied across anatomical locations; however, these results should be interpreted cautiously because several anatomical subgroups included a limited number of cases. Within the limitations of this single-center exploratory study including the relatively small sample size, heterogeneous lesion distribution, absence of external validation, and evolving LLM systems, these findings suggest that multimodal AI models may have potential as supportive tools in oral medicine. At present, they should be considered adjuncts to, rather than replacements for, clinical examination and professional judgment. Future multicenter studies using larger, more diverse, and externally validated datasets are needed to determine the reproducibility, generalizability, and clinical applicability of these findings.

## Figures and Tables

**Table 1:** Demographic and clinical characteristics of participants with normal anatomical variants.

Characteristic	Overall	Tongue	Buccal Mucosa	Lips	Vestibular Mucosa	Palate	Gingiva	Floor of the Mouth
Normal anatomical variants, n	21	11	6	1	1	1	1	0
Age (years), mean ± SD	36.3 ± 20.0	30.5 ± 16.3	34.3 ± 19.5	68.0	22.0	71.0	61.0	-
Male, n	8	4	3	1	0	0	0	0
Female, n	13	7	3	0	1	1	1	0

Data are presented as frequencies or arithmetic means ± standard deviations. Abbreviation: SD, standard deviation.

**Table 2:** Demographic and clinical characteristics of participants with pathological oral mucosal lesions.

Characteristic	Overall	Tongue	Buccal Mucosa	Lips	Vestibular Mucosa	Palate	Gingiva	Floor of the Mouth
Pathological lesions, n	49	8	8	10	3	7	11	2
Age (years), mean ± SD	52.6 ± 19.2	46.5 ± 18.1	55.5 ± 23.8	46.8 ± 20.8	48.0 ± 21.2	62.6 ± 16.2	55.0 ± 17.2	54.0 ± 24.0
Male, n	25	5	4	6	1	4	5	0
Female, n	24	3	4	4	2	3	6	2

Data are presented as frequencies or arithmetic means ± standard deviations. Abbreviation: SD, standard deviation.

**Table 3:** Top-1 diagnostic accuracy of ChatGPT-4o and ChatGPT-5 for normal anatomical variants by anatomical site.

Anatomical Site	N	Image-Only		Image-Plus-Text	
		ChatGPT-5	ChatGPT-4o	ChatGPT-5	ChatGPT-4o
Overall	21	3 (14.3%)	3 (14.3%)	16 (76.2%)	17 (81.0%)
Tongue	11	3 (27.3%)	3 (27.3%)	10 (90.9%)	10 (90.9%)
Buccal mucosa	6	0	0	4 (66.7%)	5 (83.3%)
Lips	1	0	0	1 (100.0%)	1 (100.0%)
Vestibular mucosa	1	0	0	0	0
Palate	1	0	0	0	0
Gingiva	1	0	0	1 (100.0%)	1 (100.0%)

Data are presented as frequency (%). For anatomical sites with very small sample sizes, interpret percentages cautiously because the exact confidence intervals (CI) are wide and the estimates are statistically unstable.

**Table 4:** Top-1 diagnostic accuracy of ChatGPT-4o and ChatGPT-5 for pathological oral mucosal lesions by anatomical site.

Anatomical Site	N	Image-Only		Image-Plus-Text	
		ChatGPT-5	ChatGPT-4o	ChatGPT-5	ChatGPT-4o
Overall	49	3 (6.1%)	10 (20.4%)	20 (40.8%)	31 (63.3%)
Tongue	8	0	1 (12.5%)	3 (37.5%)	3 (37.5%)
Buccal mucosa	8	0	0	3 (37.5%)	5 (62.5%)
Lips	10	3 (30.0%)	4 (40.0%)	5 (50.0%)	8 (80.0%)
Vestibular mucosa	3	0	1 (33.3%)	2 (66.7%)	2 (66.7%)
Palate	7	0	1 (14.3%)	3 (42.9%)	5 (71.4%)
Gingiva	11	0	1 (9.1%)	3 (27.3%)	6 (54.5%)
Floor of the mouth	2	0	2 (100%)	1 (50.0%)	2 (100%)

Data are presented as frequency (%). For anatomical sites with very small sample sizes, interpret percentages cautiously because the exact confidence intervals (CI) are wide and the estimates are statistically unstable.

## Data Availability

The data analysed in this study are available upon reasonable request by email to the corresponding author.

## Supplementary Materials

The following supporting information can be downloaded at: https://www.mdpi.com/article/10.3390/medicina62081610/s1, Table S1: Distribution of normal anatomical variants and pathological oral mucosal lesions included in the study; Table S2: Clinical and etiological classification of pathological oral mucosal lesions with histopathological confirmation.

## Abbreviations

The following abbreviations are used in this manuscript:

AI	Artificial intelligence
HPE	Histopathological examination
CBCT	Cone-beam computed tomography
CI	Confidence interval
CT	Computed tomography
LLM	Large language model
GLOBOCAN	Global Cancer Observatory
IARC	International Agency for Research on Cancer
STARD-AI	Standards for Reporting Diagnostic Accuracy Studies—Artificial Intelligence
OLP	Oral lichen planus
LAD	Linear IgA disease
OSCC	Oral squamous cell carcinoma
PV	Pemphigus vulgaris
BP	Bullous pemphigoid
API	Application Programming Interface
