# Supplementary material for: Artificial Intelligence for Diagnosing Normal Anatomical Variants and Pathological Oral Mucosal Lesions: A Prospective Observational Study
Source: Medicina (Kaunas). 2026 Aug 21;62(8):1610. doi: 10.3390/medicina62081610 (PMC13515991; doi:10.3390/medicina62081610)
Supplement: Supplementary file 1 [file medicina-62-01610-s001.zip › medicina-4456555-supplementary.pdf]

Table S1. Distribution of normal anatomical variants and pathological oral mucosal lesions included in the study.

Category	Diagnosis	n	% of all lesions (n = 70)
Normal anatomical variants	Geographic tongue (lingua geographica)	4	5.7
	Leukoedema	1	1.4
	Fordyce spots	3	4.3
	White sponge nevus	5	7.1
	Frenulum tag	1	1.4
	Ankyloglossia	2	2.9
	Hairy tongue (lingua villosa alba)	2	2.9
	Linea alba	1	1.4
	Palatal pits (foveolae palatinae)	1	1.4
	Torus	1	1.4
Total normal anatomical variants		21	30.0
Pathological	Oral lichen planus (OLP)	18	25.7
	Hyperkeratosis e frictione	1	1.4
	Pigmentation	1	1.4
	Minor aphthae	2	2.9
	Median rhomboid glossitis	1	1.4
	Linear IgA disease (LAD)	1	1.4
	Oral squamous cell carcinoma (OSCC)	2	2.9
	Pemphigus vulgaris (PV)	8	11.4
	Acute pseudomembranous candidiasis	1	1.4
	Chronic hyperplastic candidiasis	1	1.4
	Fibroma	1	1.4
	Bullous pemphigoid (BP)	1	1.4
	Prosthetic stomatitis	2	2.9
	Nicotine melanosis	1	1.4
	Drug-induced gingival hyperplasia	1	1.4
	Mucocele	1	1.4
	Hemangioma	1	1.4
	Nicotine stomatitis	1	1.4
	Lichenoid reaction (amalgam-associated)	2	2.9
	Crohn's disease manifestations (angioedema, tissue tags)	2	2.9
Total pathological		49	70.0
Total		70	100.0

Table S2. Clinical and etiological classification of pathological oral mucosal lesions with histopathological confirmation.

Diagnosis	n	% of all pathological lesions (n = 49)	Clinical behavior	Etiology	Histopathologically confirmed, n
Oral lichen planus (OLP)	18	36.7	Potentially malignant	Immune-mediated	17
Hyperkeratosis e frictione	1	2.0	Benign	Reactive	0
Pigmentation	1	2.0	Benign	Other	1
Minor aphthae	2	4.1	Benign	Inflammatory	0
Median rhomboid glossitis	1	2.0	Benign	Infectious	0
Linear IgA disease (LAD)	1	2.0	Benign	Autoimmune	1
Oral squamous cell carcinoma (OSCC)	2	4.1	Malignant	Neoplastic	2
Pemphigus vulgaris (PV)	8	16.3	Benign	Autoimmune	8
Acute pseudomembranous candidiasis	1	2.0	Benign	Infectious	0
Chronic hyperplastic candidiasis	1	2.0	Potentially malignant	Infectious	0
Fibroma	1	2.0	Benign	Reactive	0
Bullous pemphigoid (BP)	1	2.0	Benign	Autoimmune	1
Prosthetic stomatitis	2	4.1	Benign	Infectious	0
Nicotine melanosis	1	2.0	Benign	Other	0
Drug-induced gingival hyperplasia	1	2.0	Benign	Drug-induced	0
Mucocele	1	2.0	Benign	Reactive	0
Hemangioma	1	2.0	Benign	Vascular	0
Nicotine stomatitis	1	2.0	Benign	Reactive	0
Lichenoid reaction (amalgam-associated)	2	4.1	Benign	Inflammatory	0
Crohn's disease (angioedema, tissue tags)	2	4.1	Benign	Inflammatory	0
Total	49	100.0			30

Of the 49 pathological oral mucosal lesions, 30 (61.2%) underwent biopsy and were histopathologically confirmed. Histopathological confirmation was obtained for 17 of 18 cases of oral lichen planus (OLP), 1 case of pigmentation, 1 case of linear IgA disease (LAD), 2 cases of oral squamous cell carcinoma (OSCC), 8 cases of pemphigus vulgaris (PV), and 1 case of bullous pemphigoid (BP). The remaining 19 pathological oral mucosal lesions were diagnosed clinically on the basis of conventional examination and established diagnostic criteria. The etiologic classification included inflammatory, infectious, autoimmune or immune-mediated, neoplastic, reactive, vascular, drug-induced, and other categories, as appropriate for each diagnosis.
